# Beneficial effects of a gait used while wearing a kimono to decrease the knee adduction moment in healthy adults

**DOI:** 10.1371/journal.pone.0179260

**Published:** 2017-06-22

**Authors:** Susumu Ota, Yukari Ogawa, Hiroki Ota, Tomoya Fujiwara, Tadashi Sugiyama, Akira Ochi

**Affiliations:** 1 Department of Rehabilitation and Care, Seijoh University, Tokai, Japan; 2 Department of Physical Medicine and Rehabilitation, Nagoya Tokushukai General Hospital, Kasugai, Japan; 3 Rehabilitation Center, Yachiyo Hospital, Anjo, Japan; 4 Department of Rehabilitation Medicine, National Center for Geriatrics and Gerontology, Obu, Japan; Tokai University, JAPAN

## Abstract

The knee adduction moment (KAM) relates to medial knee osteoarthritis (OA). Several gait modifications to reduce the KAM for the prevention of knee OA have been studied. Most of the modifications, however, involve voluntary changes in leg alignment. Here we investigated the biomechanical effects for reducing the KAM of a walking style with a small trunk rotation and arm swing gait, which is a natural walking style used while wearing a kimono (Nanba walk) that shifts the ground reaction force toward the stance leg (reduced lever arm). Twenty-nine healthy adults (21.5 ± 0.6 years) participated in the present study. A three-dimensional analysis system with 10 cameras and 1 force plate was used to obtain the KAM and other biomechanical data. Surface electromyography (EMG) of the hip and trunk muscles (internal obliquus abdominal muscle: IO, external obliquus abdominal muscle: EO, multifidus muscle: MF, and gluteus medius muscle: Gmed) was also assessed, and integrated EMG (iEMG) of the four muscles was assessed in the first and second halves of the stance phase. The 1^st^ and 2^nd^ peak KAMs were significantly decreased during Nanba walking (0.40±0.09 and 0.37±0.13 Nm/kg) compared with normal walking (0.45±0.09 and 0.45±0.13 Nm/kg; P = 0.002, P<0.001, respectively). The lever arm lengths at the 1^st^ and 2^nd^ peak KAMs were also significantly decreased during Nanba walking compared with normal walking (p = 0.023 and p<0.001, respectively). The iEMGs of IO, EO and Gmed muscles during the first half, and the iEMGs of EO and GM during the second half of the stance phase were significantly increased during Nanba walking compared with normal walking. The Nanba gait modification could be a useful strategy for reducing the KAM with high activation of the trunk and hip joint muscles for the prevention and/or treatment of medial knee OA.

## Introduction

The knee adduction moment (KAM) during walking predicts the progression of medial knee osteoarthritis (OA) [1, 2]. Several gait modifications to reduce the KAM have been studied to prevent knee OA. Previous studies described gaits with a slow speed [3, 4], toe-out gait to increase the foot progression angle [5, 6], toe-in gait to decrease the foot progression angle [7, 8], increasing the ipsilateral trunk lean (sway) [9, 10], medial thrust with slight knee flexion and medialization of the knee of the stance leg, increasing the hip internal abduction moment strategy [11] as modifications to reduce the KAM. These gait modifications without slow speed walking biomechanically shorten the lever arm of the KAM on the frontal plane. Toe-out and trunk lean gait modifications are often observed as compensatory adjustments in patients with knee OA, but the application of these modifications to prevent the progression of knee OA in patients without knee pain, such as those with early knee OA, might not be comfortable to implement. Shull et al. [12] reported gait retraining with a toe-in gait and trunk sway using real-time feedback. Subjects could freely select only one kinematic change, either the toe-in gait or trunk sway. The trunk lean modification induced negative side effects, including discomfort, difficulty maintaining posture, and decreased balance. In addition, whether those gait modifications should be recommended as long-term interventions for healthy individuals or for patients with early-stage knee OA remains questionable, because, as mentioned above, these gait modifications are often observed as compensatory modifications in patients that already have knee OA. Other studies to prevent knee OA report that weakness of the hip abductor muscle [13] and an increased knee flexion moment during the gait [14] are related to medial knee OA.

The traditional Japanese walking style used while wearing a kimono, sometimes called Nanba style walking (Nanba walk), features small trunk rotation movements with a natural small arm swing that results from placing the hands on top of one another on the belly or placing the hands over the groin without constraining the arm swing, and the swinging arm moving forward during the forward movement of the swinging leg (i.e., the arm and leg on the same side move in the same direction) [15–17]. Observations suggest that the Nanba walk synchronizes the rotation of the pelvis and trunk, with the upper body moving smoothly above the supporting limb as when walking on stilts [17, 18]. Thus, the ground reaction force during the Nanba walk is assumed to shift toward the stance leg, and the shortened lever decreases the KAM. This is based only on observation, however, because few biomechanical studies have examined the effects of this gait modification.

The present study aimed to investigate the biomechanical effects of the Nanba walk for reducing the KAM and its effects on other biomechanical values, such as the knee flexion moment and muscle activities of the hip joints, compared to normal walking. Additionally, because trunk muscle activity is thought to be higher during Nanba walking than during normal walking to restrict the trunk rotation, we also investigated the trunk muscle activity as a feature of Nanba walking.

## Methods

### Participants

Thirty healthy adults participated in the present study and their gaits were analyzed. After processing the data, however, we found that one participant did not fulfill the walking speed criteria and the data for this subject were therefore excluded from the final analysis. Finally, twenty-nine healthy adults (16 men, 13 women) with a mean age of 21.5 ± 0.6 years (range, 20–22), mean height of 165.4 ± 10.1 cm, and mean body mass of 56.1 ± 9.9 kg participated in the present study (Table 1, S1 Table). Subjects were recruited from the student body at the Department of Rehabilitation and Care of Seijoh University using a leaflet and poster on a bulletin board from August through November in 2015.

**Table 1:** Subject characteristics. Please refer to the supplemental data for details.

Sex	Male 16/ Female 13
Age [years], Range	21.5±0.6, 20–22
Body height [cm]	165.4±10.1
Body mass [kg]	56.1±9.9
Measured leg	Right 14/ Left 15

### Ethics statement

The Ethics Committee of Seijoh University approved the study, and all subjects provided written informed consent to participate in the study.

### Three-dimensional gait analysis

**Fig 1:**
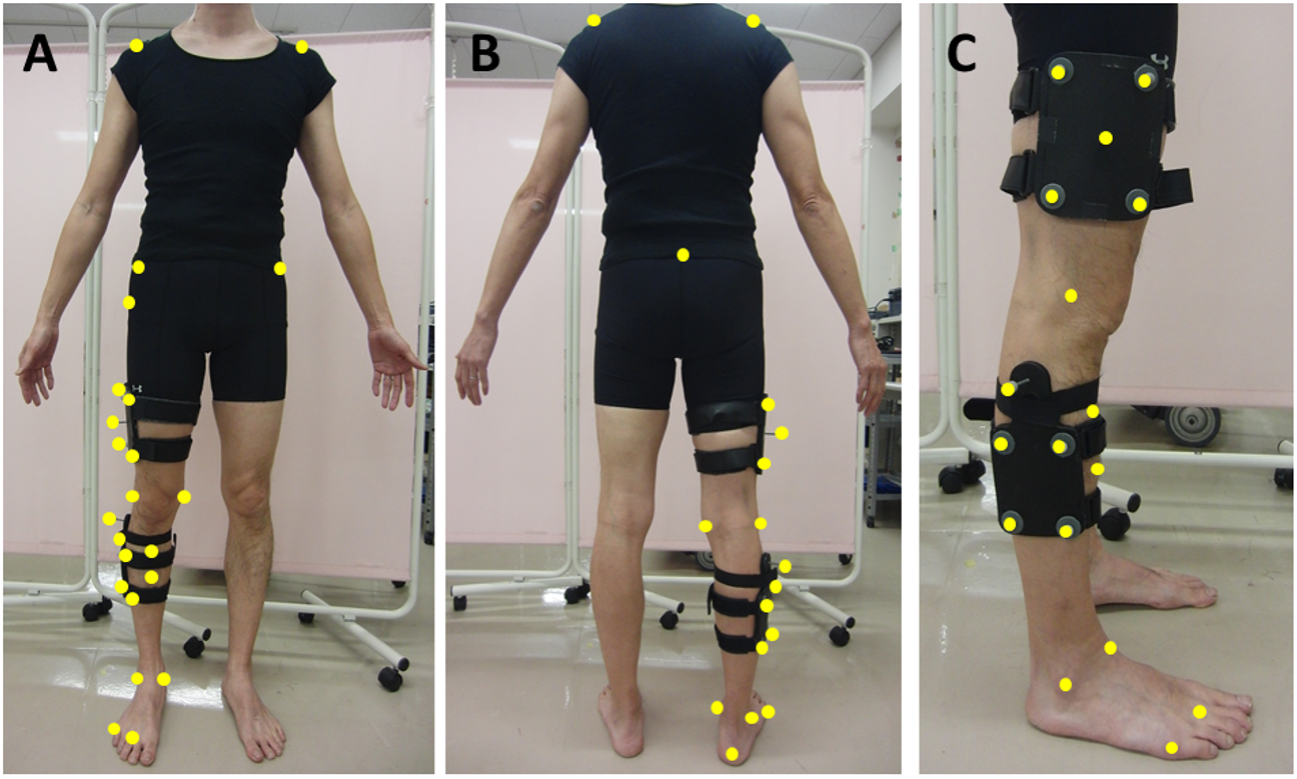
Marker set for static calibration. Fig 1-A, B, C shows the frontal, back, and sagittal views, respectively, for measuring a right leg. The measured leg was randomly selected. The markers on the medial epicondyle and medial malleolus were removed during the gait task.

**Fig 2:**
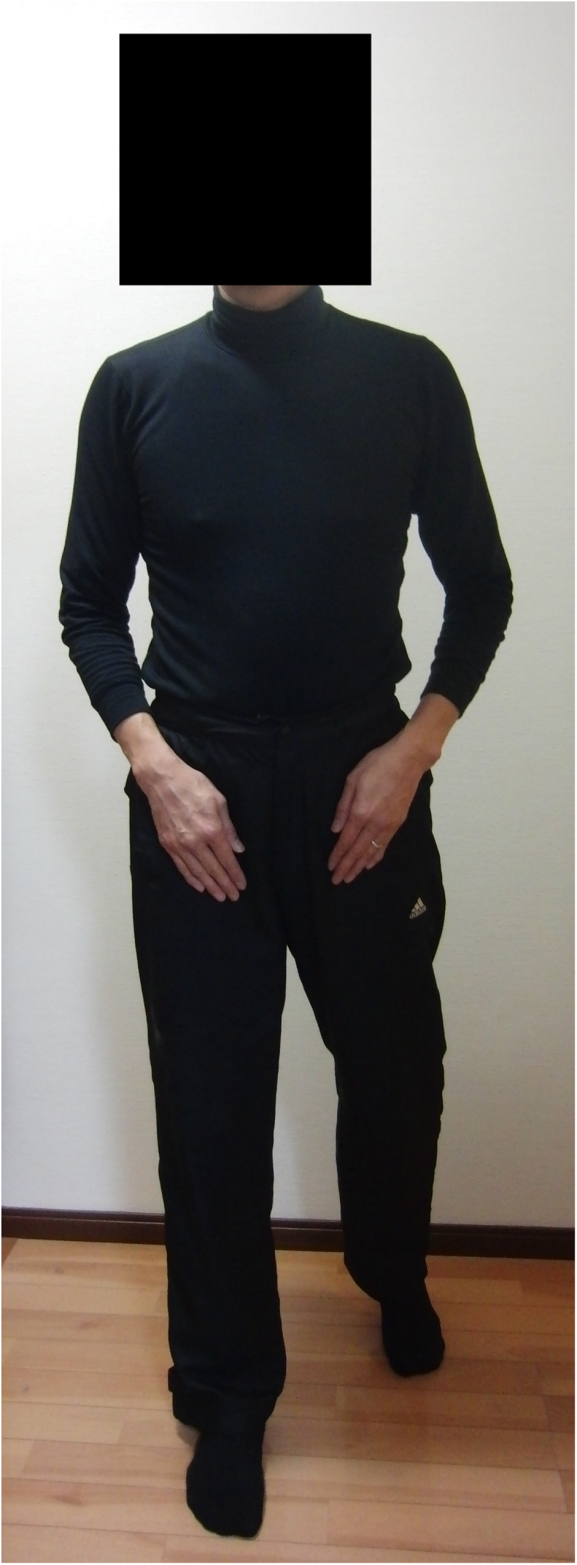
Procedure for the Nanba walk. Placing the hands over the groin is an easy way to perform the Nanba walk.

The gait was evaluated using three-dimensional (3D) gait analysis and the kinetic and kinematic values of the gait were determined using previously reported methods [19]. The 3D trajectory data, sampled at 100 Hz, were collected using a 10-camera motion analysis system (Motive; Optitrack Japan, Tokyo Japan; Venus 3D; Nobby Tech, Tokyo, Japan) and digitally recorded. A force plate (AccuGait; AMTI, MA, USA) was used to measure reaction forces at a rate of 100 Hz. The data from the force plate and 3D motion analysis system were synchronized. We placed 25 reflective sphere markers (7-mm diameter) at various body sites, and cluster markers on the thigh and lower leg (Fig 1). The measured leg (right or left) was randomly selected. Subjects performed the tests while barefoot, and were first asked to walk using their usual normal walking style. The subjects were then asked to walk using the Nanba walking style within ±5% of their self-selected speed during normal walking. The walking speed was confirmed by measuring the movement of the second sacrum marker. While walking using the Nanba gait, subjects were asked to place their hands over the groin area (Fig 2). The subjects walked along a 6-m walkway, and three successful trials were recorded for each subject. Walking speed (m/s) and stride length (m) were also measured as the temporal-spatial values.

A four-link model with four segments for the pelvis, thigh, shank (lower leg), and foot was used in the present study, as reported previously [19]. Global optimization was used to estimate the segments [20], and the model was customized to each subject using the marker data collected during each individual’s static calibration. These marker coordinates were used to define segment-embedded reference frames for the associated body parts [21]. We assessed the inertial properties of each limb segment based on the Japanese inertial characteristics [22]. The hip joint center was defined using the Davis method [23] based on the anterior superior iliac spine and posterior superior iliac spine. The center of the knee joint was defined as the midpoint between the lateral and medial femoral epicondyle, and ankle joint centers were defined as the midpoint between the medial and lateral malleolus.

External KAM was calculated using inverse dynamics. The KAM was normalized to the body mass (Unit: Nm/kg). KAM measurements from the three trials were averaged for the analysis. All data were normalized to 100% of the stance time, where 0% is the heel strike of the measured leg and the 1^st^ and 2^nd^ peaks of KAM during the stance phase were determined. The lengths of the frontal plane lever arms at the 1^st^ and 2^nd^ peaks of the KAM were measured as the perpendicular distance between the resultant frontal plane ground reaction force line of action and the center of the knee joint.

In addition, the peak knee flexion moment (also normalized to body mass: Nm/kg) and peak knee flexion angle during mid-stance (18%-50% of the stance phase) [24] were measured. The trunk rotation angle was measured as the angle between the lines connecting the bilateral acromion markers and the anterior superior iliac spine on the horizontal plane. Positive values indicate a normal gait pattern at initial contact; i.e., when the initial contact is made with the right leg, the right anterior superior iliac spine moves forward and the right acromion backward. For the left leg, positive values indicate the same pattern as for the right leg. The peak (maximum) values of trunk rotation during the first half of the stance phase and the peak (minimum) values of the rotation during the second half of the stance phase were used in the final analysis.

### Surface electromyography of hip and trunk muscles

Surface EMG of the trunk and hip muscles on the measured leg side was acquired to determine the activities of the internal obliquus abdominal muscle (IO), external obliquus abdominal muscle (EO), and multifidus muscle (MF), which are thought to relate to trunk stability, and the gluteus medius muscle (Gmed) which is thought to relate to KAM [11, 25]. Surface EMG of those muscles was obtained using disposable silver/silver chloride surface electrodes with a 1-cm recording diameter (Blue Sensor M-00-S, Ambu Corp, Copenhagen, Denmark). Bipolar electrode pairs and a ground electrode were placed longitudinally over the muscle at 2.5-cm intervals after preparing the skin. Electrodes were placed at the IO, EO, MF, and Gmed acording to standard instructions (i.e., SENIAM recommendations [26] and Cram’s instructions [27]). EMG signals were recorded on each muscle using an EMG acquisition system (Mwatch; Wada Aircraft Technology Co., Ltd., Kiyosu, Japan). The EMG signals were sampled at 1000 Hz, amplified and band-pass filtered (10–300 Hz), and then rectified. Integrated EMG (iEMG: mVS) of four muscles in the first half and second half of the stance phase were used for the final analysis.

### Data analysis

The data were tested for normal distribution using the Shapiro-Wilk test. For normally distributed data, the paired t-test was used to determine differences in the biomechanical values between the normal and Nanba walking conditions. For non-normally distributed data, the Wilcoxon signed-rank test was applied. A *p* value less than 0.05 was considered to indicate significance, and effect size (Choen’s d) was also evaluated in all comparisons. All statistical analyses were performed with SPSS, Version 16.0 (IBM Japan, Chuo Ward, Tokyo, Japan).

## Results

**Fig 3:**
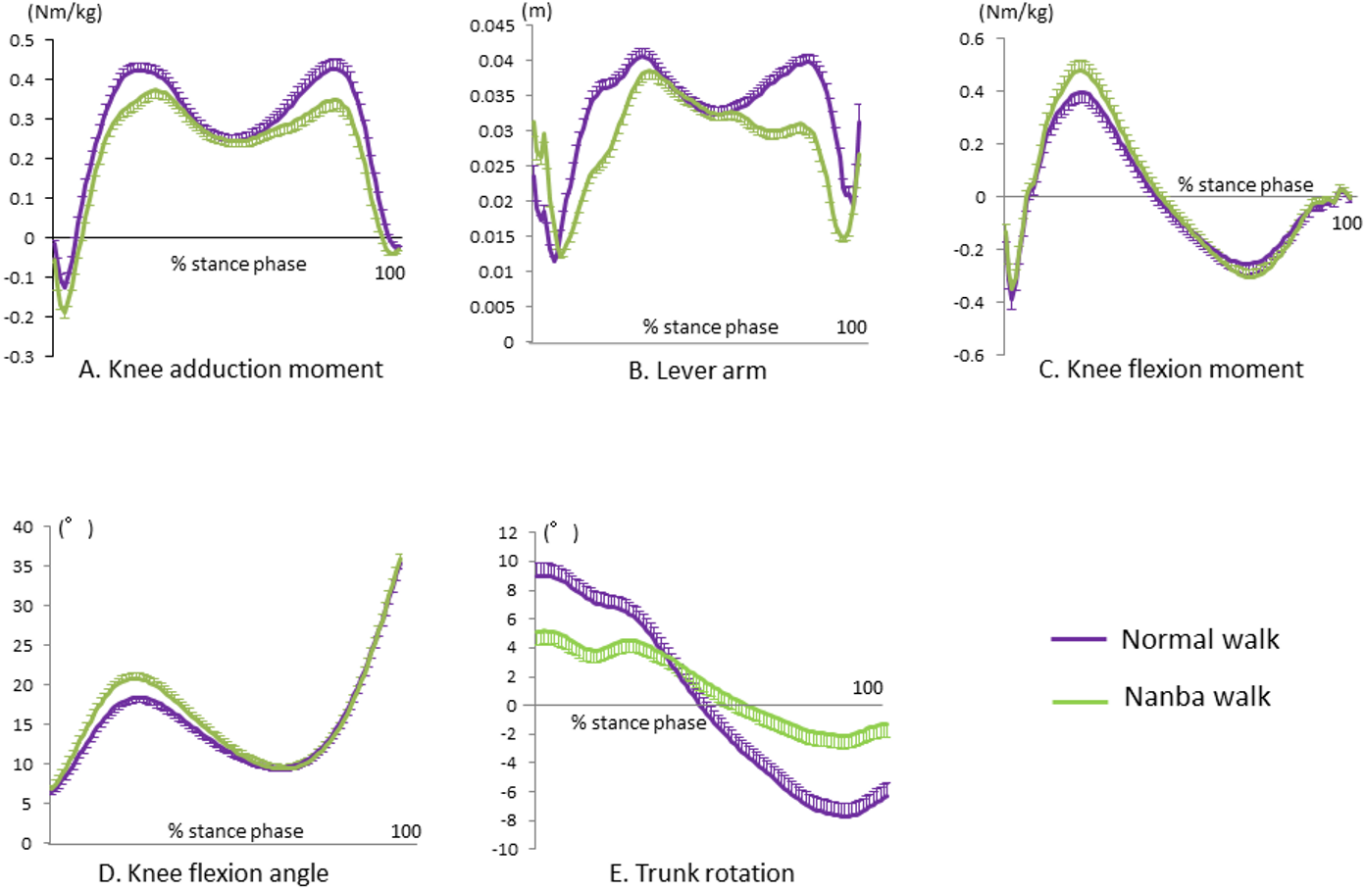
Knee kinematics and kinetics with standard error bars during the stance phase.

Table 2 (S2 Table), and Fig 3 show the biomechanical results with comparison of both gaits. The gait speed, stride length, and 1^st^ and 2^nd^ vertical ground reaction force were not significantly different between the two gait conditions.

**Table 2:** Comparison of biomechanical data between normal walking and Nanba walking with a small arm swing.

	Normal walk	Nanba walk	P-value	ES
**Gait speed (m/s)**	1.3±0.1	1.3±0.1	0.285^a^	0.13
**Step length (m)**	1.31±0.11	1.32±0.11	0.283^b^	0.07
**1**^**st**^ **Vertical GRF (N)**	623.6±114.5	631.0±112.3	0.198^a^	0.07
**2**^**nd**^ **Vertical GRF (N)**	628.9±116.5	637.0±127.8	0.107^a^	0.07
**1**^**st**^ **KAM (Nm/kg)**	0.45±0.09	0.40±0.09	0.002^b^	0.60
**2**^**nd**^ **KAM (Nm/kg)**	0.45±0.13	0.37±0.13	<0.001^b^	0.55
**1**^**st**^ **LA at peak 1**^**st**^ **KAM (m)**	0.039±0.012	0.036±0.013	0.023^b^	0.28
**2**^**nd**^ **LA at peak 2**^**nd**^ **KAM (m)**	0.040±0.010	0.033±0.010	<0.001^b^	0.71
**KFM (Nm/kg)**	0.41±0.23	0.50±0.21	<0.001^b^	0.40
**Knee flexion angle (°)**	18.6±6.5	19.2±6.1	0.358^a^	0.10
**1**^**st**^ **Trunk rotation angle (°)**	9.2±4.5	5.7±4.7	<0.001^c^	0.77
**2**^**nd**^ **Trunk rotation angle (°)**	-8.0±4.8	-2.6±4.8	<0.001^b^	1.12

ES = effect size; GRF = ground reaction force; KAM, knee adduction moment; LA, lever arm; KFM, knee flexion moment

^a^ Wilcoxon signed-rank test

^b^ paired t-test

The 1^st^ and 2^nd^ peak KAM during Nanba walking were significantly decreased compared to the KAMs during normal walking (Table 2). The lever arm lengths at both peak KAMs were also significantly decreased compared with the lengths during normal walking (Table 2). The peak knee flexion moment during Nanba walking was significantly increased compared with that during normal walking, but not the knee flexion angle (Table 2). The 1^st^ and 2^nd^ peak trunk rotation, based on the difference between the angles of the lines from the pelvis to both acromions, was significantly decreased during Nanba walking (5.7±4.7, -2.6±4.8°) compared with that during normal walking (9.2±4.5, -8.0±4.8°).

For the muscles activities, iEMGs of IO, EO, and Gmed during the first half of the stance phase and iEMGs of EO and Gmed during the second half of the stance phase were significantly increased during Nanba walking compared with normal walking (Table 3, S3 Table).

**Table 3:** Comparison of the iEMG of the trunk and hip muscles during the first and second halves of the stance phase.

(Unit: mVS)	Normal walk	Nanba walk	P-value	ES
**First half of stance phase**				
**IO**	2.89 ± 1.70	3.12 ± 1.51	0.017^a^	0.14
**EO**	3.10 ± 1.33	4.08 ± 1.74	<0.001^a^	0.63
**GM**	2.72 ± 1.31	2.99 ± 1.43	0.008^b^	0.19
**MF**	4.30 ± 1.79	4.52 ± 1.75	0.057^b^	0.13
**Second half of stance phase**				
**IO**	2.58 ± 1.51	2.45 ± 1.36	0.905^a^	0.09
**EO**	2.42 ± 1.00	2.69 ± 0.97	0.014^a^	0.27
**GM**	1.87 ± 0.92	2.04 ± 1.04	0.048^b^	0.18
**MF**	3.27 ± 1.43	3.49 ± 1.42	0.144^b^	0.16

ES, effect size; IO, internal oblique abdominal muscle; EO, external oblique abdominal muscle; GM, gluteus medius muscle; MF, multifidus muscle

^a^ Wilcoxon signed-rank test

^b^ paired t-test

## Discussion

**Fig 4:**
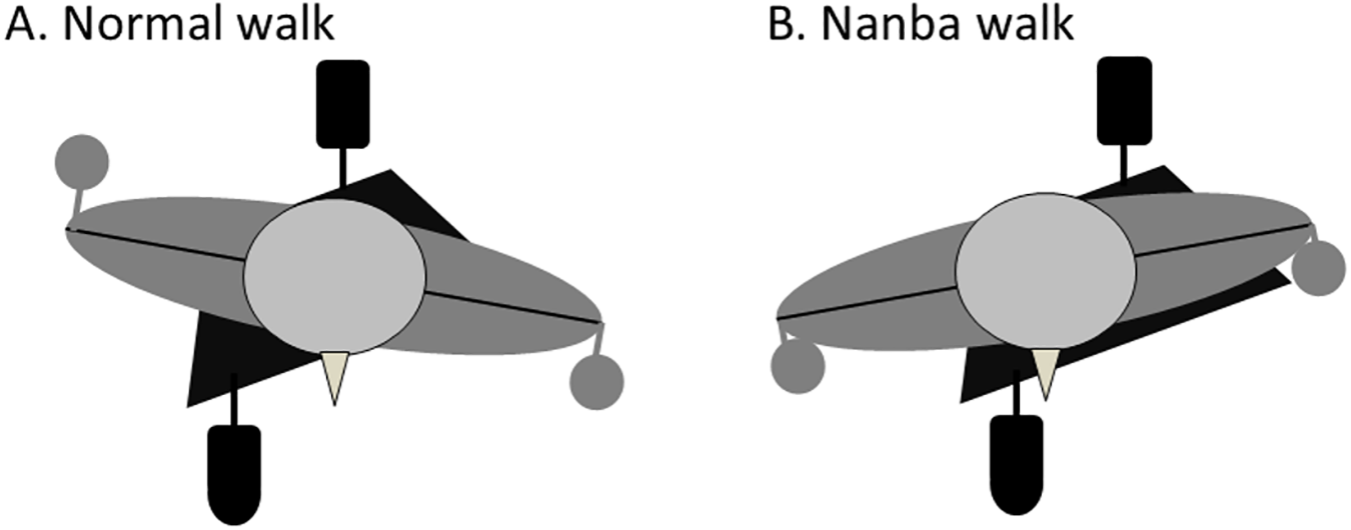
Schema of the normal walk and the Nanba walk from the top view. The difference in the angle (trunk rotation) between the pelvis (both anterior superior iliac spines) and both acromion lines during Nanba walking is smaller than that during normal walking because the arm and leg on the same side move in the same direction. The upper body is assumed to shift laterally toward the stance limb in the frontal plane.

The present study evaluated the effect of altering the gait to modify the KAM. Nanba walking significantly decreased the 1^st^ and 2^nd^ peak KAMs compared with normal walking. The mean percent decrease in the 1^st^ KAM was 11%. Several studies have reported gait modifications to reduce the KAM. For example, walking 15% slower than a self-selected walking speed decreases the 1^st^ KAM by 8% [4]; KAM is reduced by 9% by adding a 6° trunk lean during walking compared with a normal gait [9]; and the use of a cane decreases the KAM by 10% [28]. A recent review [29] indicated that a toe-out gait has a wide range of positive and negative effects on the KAM (ranging from a 55.2% decrease to a 12.7% increase) [30], and therefore the effects of the toe-out gait for reducing the KAM are not clear. Gait modifications may produce a wide range of beneficial effects for the 1^st^ KAM. The decreased KAM produced by Nanba walking, however, might not be smaller than that produced by those gait modifications without a toe-out gait. The decreased KAM is assumed to be due mainly to shortening of the lever arm of KAM, and, in the present study, the lever arm length at the 1^st^ and 2^nd^ peak KAM with Nanba walking was significantly shorter than that during normal walking (Table 2 and Fig 3B). The vertical ground reaction force, walking speed, and stride length were not significantly different between the two walking styles. The lever arm was shorter during Nanba walking, which means the ground reaction force shifts toward the stance leg (knee joint center) on the frontal plane. Because the direction of the ground reaction force is thought to be toward the center of the mass, we assume that the center of mass (upper body) during Nanba walking is shifted laterally toward the stance foot (knee joint center). Also, as another feature of the Nanba walk, the difference in the angle between the pelvis and trunk (trunk rotation) during Nanba walking was smaller than that during normal walking, indicating that the pelvis and trunk have a similar rotation phase (Fig 3E). Fig 4 shows the schema of normal walking (Fig 4A) and Nanba walking (Fig 4B) as viewed from the top with two features: lateral shift of the center of mass and small trunk rotation. Additionally, the lever arm length in the early stance phase (from the loading response to the mid-stance phase) during Nanba walking is smaller than that during normal walking (Fig 3), and may be another feature of the Nanba gait, which should be investigated in relation to the upper body mass. We did not, however, assess the center of mass of the upper body, and the mechanism should be also analyzed in relation to the upper body mass.

Gait modifications in previous studies with voluntary alignment alterations of the leg and trunk lean have had some negative side effects, especially when applied as a long-term intervention, such as over several years. Hunt et al. [31] reported that participants with a 10-week toe-out gait modification intervention have minimal to moderate difficulty, and 33% of participants feel joint discomfort within the first 2 weeks. The authors reported no data regarding the negative side effects over the long-term. The Nanba gait used in the present study, however, is a walking style with a small trunk rotation and arm swing (placing the hands over the groin), and is the walking style that is naturally used by people wearing a kimono in Japan. Therefore, the Nanba gait might be a more natural and more easily adhered to gait modification compared with gait modifications that require alternating the legs and trunk alignment. We do not know, however, how the Nanba walk would be adhered to by those not familiar with wearing a kimono. Further studies should be performed to investigate the ability to adhere to the Nanba gait and the negative influence of a long-term intervention with subjects familiar and not familiar with wearing a kimono.

The values of the peak knee flexion moment were significantly higher during Nanba walking than during normal walking. Yang et al. [32] reported a vertical center of mass displacement when the hands were placed on the chest, similar to the conditions of the Nanba walk. They found that the center of mass displacement with the arms constrained was greater than that during normal walking, because of an inverse relationship between the direction of the arm center of mass and the direction of the body center of mass. The authors concluded that the vertical center of mass displacement without an arm swing increased due to an acceleration change in the arm center of mass. Although we obtained no arm acceleration data in the present study, Nanba walking without this arm movement (acceleration) might increase the knee flexion moment. For the knee flexion moment, the moment is associated with the medial knee compression force [33], few longitudinal and interventional studies have examined the influence on medial knee OA. The knee flexion moment during the toe-out gait is significantly higher than that during normal walking [6], and the toe-out gait is reported to have beneficial effects for patients with knee OA after a 10-week intervention [31]. Although the peak knee flexion moment has no clear influence on medial knee OA [31], a potentially negative effect of the Nanba gait is that the moment could be increased during the stance phase.

The hip (Gmed) and trunk (EO) muscles were highly activated during Nanba walking in the first and second halves of the stance phase (IO was highly activated in only first half of the stance phase). Little information regarding the activation of those muscles during Nanba walking is available. The Nanba gait might decrease pelvic excursion, although this was not examined in the present study. The high trunk muscle activation might be similar to the draw-in maneuver gait, a gait that also reduces the KAM [34]. Regarding Gmed activation, Chang et al. [35] reported that hip abduction weakness in the stance limb shifts the center of the body mass toward the swing limb, and this shift increases the KAM due to an increase in the lever arm length. The Gmed muscle is assumed to restrict the pelvic drop on the swing leg side, and could therefore prevent increases in the KAM [10]. Previous studies on strengthening the Gmed [36] and experimentally induced Gmed weakness [37], however, did not demonstrate an increase in the KAM. Strengthening or activating the Gmed alone might not be directly associated with the KAM, and, together with activation of the trunk muscles, this could be important for the decrease in the KAM observed in the present study. Future studies should investigate whether the high activation of the hip and trunk muscles leads to negative effects, such as injury or fatigue.

There are several limitations to this study. First, because the subjects were healthy individuals, the KAM of patients with knee OA (even in the early stage) should be assessed during Nanba walking. Second, arm swing is reported to relate to balance recovery [38], and thus negative side effects of the Nanba walk in older persons with knee OA should be investigated. Therefore, the arm swing should be assessed during Nanba walking. Third, this study examined only the immediate effect of the modification. The compliance rate and any consequent disorders should be investigated in a longitudinal study. In addition, the beneficial effects and adequate conditions of Nanba walking should be clarified. Fourth, the center of the upper body mass must be determined using full body markers to clarify the mechanism of reduced KAM due to the Nanba gait. Finally, the discussion is based on significant differences in the biomechanical and iEMG values between the two walking conditions, but we must consider that the effect size of some of those assessed values were small to moderate.

In conclusion, the 1^st^ and 2^nd^ peaks of the KAM during Nanba walking were significantly lower than those during normal walking due to shortening of the lever arm length of KAM. The Nanba walk features high hip and trunk muscle activity compared to normal walking. The Nanba walk modification could be a useful strategy for reducing the KAM for the prevention and/or treatment of medial knee OA.

## Supporting information

S1 Table Subject characteristics. (XLSX)

S2 Table Biomechanical and temporal-spatial values in normal and Nanba walk. (XLSX)

S3 Table Integral EMGs in normal and Nanba walk. (XLSX)
